# Persistent ascites resolving with gonadotropin-releasing-hormone-agonist 18 months after hospitalization for severe ovarian hyperstimulation syndrome

**DOI:** 10.1007/s00404-013-2940-7

**Published:** 2013-07-12

**Authors:** Cihan Comba, Funda Gungor Ugurlucan, Ercan Bastu, Ahmet Cem Iyibozkurt, Samet Topuz

**Affiliations:** Kadin Hastaliklari ve Dogum Anabilim Dalı, Istanbul Universitesi Istanbul Tip Fakultesi, Capa Findikzade, 34093 Istanbul, Turkey

**Keywords:** Ovarian hyperstimulation syndrome, Ascites, Infertility, Ovarian stimulation

## Abstract

Ovarian hyperstimulation syndrome (OHSS) is a life- threatening complication of controlled ovarian stimulation. One of the main symptoms of OHSS is ascites. Treatment is symptomatic with resolution of the symptoms over days to weeks. We report a case of severe OHSS with persistent ascites 18 months after the diagnosis. Persistent ascites secondary to OHSS was diagnosed and single dose leuprolide acetate depot 11.25 mg was administered. At follow-up, no ascites was observed.

## Introduction

Ideal treatment outcome in assisted reproduction is optimum ovarian stimulation with minimal rate of severe ovarian hyperstimulation syndrome (OHSS). OHSS is a life-threatening complication of controlled ovarian stimulation and is associated with increased capillary permeability, fluid leakage into the third spaces, and hemoconcentration [1]. Treatment is symptomatic with resolution of the symptoms, hemoconcentration, and ascites over days to weeks.

We report a case of severe OHSS with persistent ascites 18 months after the diagnosis. The patient underwent full ascites work-up, but the persistent ascites was attributed to OHSS. Ascites resolved after treatment with leuprolide acetate.

## Case

A 38-year-old primiparous woman was referred from the gastroenterology unit for evaluation of persistent ascites. The patient’s gynecological examination was unremarkable. She had regular menstrual periods. Transvaginal ultrasonography revealed bilateral normal sized ovaries and moderate amount of ascites. Blood count and biochemical tests showed no pathology; hemoglobin was 12.8 g/dl, hematocrit was 36 %, platelet was 246,000/dl, and white blood cells 6,200/dl. Liver function tests were normal, albumin was 4.5 g/dl, total protein was 6.9 g/dl, protrombin time (PT) was 12.1 and international normalized ratio (INR) value was 1.06. The patient had a history of controlled ovarian stimulation with the indication of unexplained infertility and severe OHSS requiring hospitalization 18 months ago. Antagonist protocol was used, 4,200 IU recombinant follicle stimulating hormone (rFSH) was given on Days 3–15, and cetrorelix was added on Day 10 and was given for 7 days, ovulation triggering was performed with 250 μgr recombinant human chorionic gonadotropin on day 16. Twenty-nine follicles were aspirated and 18 oocytes were retrieved. Three embryos were transferred on Day 3. Six days after the embryo transfer, the patient developed signs of severe OHSS and was hospitalized. Symptomatic treatment including fluid and albumin infusion and low-molecular-weight heparin was given. Beta-hCG was positive on Day 12 and transvaginal ultrasonography revealed single 5 mm intrauterine gestational sac 1 week later. She was hospitalized for 15 days when symptoms decreased; however, she had ascites at discharge. The pregnancy continued without any complications except for persistent ascites throughout pregnancy. The patient delivered a healthy baby at the 38th gestational week by vaginal delivery.

Ten months after discharge, the patient was admitted to the emergency unit with severe abdominal pain. Abdominal ultrasonography revealed cholelithiasis and ascites. Laparoscopic cholecystectomy was performed with the diagnosis of acute biliary pancreatitis. During laparoscopy, huge quantity of abdominal free fluid was aspirated and the patient was referred to the gastroenterology unit for further investigation. Biochemical evaluation of the ascites was as follows: serum-ascites albumin gradient, <1.1; glucose, 85 mg/dl; LDH, 184 IU/L; total protein, 3.8 g/dl; albumin, 2.8 g/dl; leukocyte, 480/L and neutrophil, 170/L of the ascites have been determined. Simultaneous serum biochemical analysis revealed glucose, 85 mg/dl; LDH, 252 IU/l; total protein, 5.8 g/dl; and albumin, 3.3 g/dl. Ascites/serum ratios for bilirubin and amylase were normal. Cultures and cytology of the ascites were negative. Cardiac functions were also evaluated and echocardiography was unremarkable except for minimal tricuspid and pulmonary valve insufficiency. Abdominopelvic computerized tomography revealed increase in the size of liver and spleen, ascites, extensive contrast enhancement and thickening of the peritoneum. Gastroscopy, colonoscopy, and portal system Doppler ultrasonography were performed to exclude hepatic and portal system pathology and no abnormality was found. Antiparietal cell and anti-intrinsic factor antibodies were negative. Persistent ascites secondary to OHSS was diagnosed and single dose leuprolide acetate depot 11.25 mg was administered. At the follow-up examination 2 months later, no ascites was observed at the transvaginal ultrasonography. No complications developed during follow-up and the ascites did not recur at the end of 3 months.

## Discussion

Ovarian hyperstimulation syndrome may develop as a result of controlled ovarian stimulation in assisted reproduction and is the most severe complication of this treatment [2]. The main features of OHSS are abdominal pain, nausea, vomiting, ascites, distention, localized or generalized peritonitis, acute abdominal pain, hypotension and/or hypovolemia, dyspnea, electrolyte imbalance, and acute renal failure. Ascites may be so severe that paracentesis may be necessary to relieve respiratory problems [3]. Treatment includes hospitalization, fluid therapy, and thromboprophylaxis.

The ultimate pathophysiological step underlying this clinical situation is increased vascular permeability. With the administration of hCG for ovulation triggering, the expression vascular endothelial growth factor (VEGF) and VEGF receptor-2 (VEGFR-2) mRNA increase significantly rising to a maximum coinciding with peaked vascular permeability [4]. Inflammatory cytokines have been shown to be elevated in the ascites fluid of patients with OHSS [4].

Leuprolide acetate is a gonadotropin releasing hormone agonist (GnRH-a) used for assisted reproduction and other indications in gynecologic practice. Gonadotropin-releasing hormone agonists have decreased luteotropic effects when compared with human chorionic gonadotropin and have been shown to be effective in preventing OHSS in high-risk patients [5–10]. Kitajima et al. [11] administered GnRH-a to hyperstimulated rats and measured the concentrations of VEGF, VEGF receptors, and vascular permeability. They found that GnRH-a treatment significantly reduced expressions of VEGF, VEGFR-1, and VEGFR-2 both in mRNA and protein levels in the ovaries of hyperstimulated rats. GnRH-a treatment also reduced vascular permeability in the ovaries of hyperstimulated rats. They concluded that GnRH-a treatment may prevent early OHSS by reducing vascular permeability through the decrease in VEGF and its receptors.

Major limitation of the study is the development of acute biliary pancreatitis during follow-up for ascites. Pancreatic ascites makes up less than 3 % of cases of ascites [12]. Typically, who had pancreatic ascites the ascites/serum ratio for amylase is 6:1. Our patient’s ascites may not be precisely possible to differentiate the ascites during the course of OHSS from ascites secondary to acute biliary pancreatitis. In addition, the ascites was present before, during, and after pregnancy and responded to treatment with GnRH analogue, which makes ascites secondary to OHSS more likely.

As far as we know, our case is the first in the literature about persistence of ascites longtime after hospitalization for OHSS. Ascites had persisted for 18 months despite normal blood count and biochemical tests and ovarian functions. Persistence of ascites may be due to long acting ovarian and peritoneal capillary permeability due to various cytokine effects. In our case, response to GnRH-a treatment hypothesizes involvement of ovaries in the regulation of long acting capillary permeability and persistent ascites long-term after OHSS. Further researches on the issue with higher number of patients from different centers are warranted to establish a diagnosis and treatment protocol.

## Conclusion

Ascites may persist in patients suffering from severe OHSS long after the biochemical values and ovarian functions normalize. GnRH-a treatment may be successful in these cases.
